# Supplementary material for: A conflict between spatial selection and evidence accumulation in area LIP
Source: Nat Commun. 2022 Aug 1;13:4463. doi: 10.1038/s41467-022-32209-z (PMC9343639; doi:10.1038/s41467-022-32209-z)
Supplement: Supplementary file 1 — Supplementary Information [file 41467_2022_32209_MOESM1_ESM.pdf]

SUPPLEMENTARY INFORMATION

A conflict between spatial selection and evidence accumulation in area LIP

Joshua A. Seideman, Terrence R. Stanford, and Emilio Salinas

Department of Neurobiology and Anatomy, Wake Forest School of Medicine, 1 Medical Center Blvd., Winston-Salem, NC 27157-1010, USA

Contents

Supplementary Note

Supplementary Figures 1–14

Supplementary References

Abbreviations

CRDM, compelled random dot motion

KHS, Kiani, Huk, and Shadlen

LIP, lateral intra-parietal

MD, motion duration

RDM, random dot motion

RF, response field

rPT, raw processing time

RT, reaction time

SD, standard deviation

SE, standard error

Supplementary Note

On the surface, our analyses may seem similar to previous efforts to chart choice accuracy as a function of time[1,2,3], but there is a fundamental difference between processing time (rPT) in our urgent tasks and other time measures used in prior studies (RT, stimulus duration, estimated decision time, etc.). Based on rPT, the evolution of the perceptual judgment can be tracked with an effective temporal resolution of one to a few milliseconds[4] — versus hundreds of milliseconds with traditional methods.

As a point of comparison, consider the study[3] by Kiani, Huk, and Shadlen (KHS) in which subjects performed the standard RDM task but the dot motion was presented for limited amounts of time, between 80 and 1500 ms. As expected, accuracy increased steadily with motion duration (MD). However, beyond such qualitative relationship, it is essentially impossible to extract from their data a reliable estimate of the time at which the perceptual discrimination was completed.

First note that, with an MD of 100 ms, the 'packet' of sensory evidence has much more than 100 ms to circulate within the decision-making circuitry. In the KHS experiment, the go signal coincided with the termination of the motion stimulus, and the RT was counted from the onset of the dots. So, suppose that a stimulus of 100 ms duration was presented at $t = 0$ and the monkey responded to it at $t = 400$ ms (so the RT was 400 ms, a typical value in the KHS study). The processing time is not 100 ms, which in our urgent-choice framework would definitely correspond to a guess, because the stimulus had 300 ms of additional time to be processed after its offset. It is unclear how much rPT this 100+300 ms combination actually corresponds to, because it is unknown how MD and 'packet' circulation time each contribute to the perceptual judgment, but our data suggest that the effective rPT is probably closer to the maximum 400 ms, given the high performance levels achieved at all coherences in the KHS experiment (their Fig. 3A).

Critically, the difference is not a matter of a simple, constant offset. In a large sample of 100+c ms trials, the circulation time c will vary widely, given the typical spread in RTs. So, on average, this one condition (MD = 100 ms) will include choices with a wide variety of underlying sensory-motor dynamics. The same is true for other MD values. As a consequence, the curve of accuracy versus MD varies very slowly, and does not unambiguously indicate when the perceptual evaluation was made. Now, here is the fundamental distinction: **our urgent tasks enforce a circulation time equal to zero**. This eliminates an enormous amount of variance, and leads to a curve of accuracy versus rPT, the tachometric curve, that varies sharply and is not offset, so it veridically tells us the probability of the perceptual evaluation having beeen completed at each point in time — information that cannot be derived through traditional analyses.

Consider. In the KHS experiment (their Fig. 3A), with an MD of 100 ms, accuracy was already above 95% correct at 51% coherence, so the MD needed to reach 75% correct at 100% coherence could be easily extrapolated to 50 ms or less. But it would make no sense to interpret any of these numbers as a decision time or temporal threshold: visual latencies consume at least 50 ms, and in the traditional dots task, LIP and other oculomotor areas do not start differentiating until at least 200 ms after stimulus onset, according to Fig. 2c, d and numerous reports[3,5,6,7], including KHS (their Fig. 2A). If the neurons that supposedly guide the choice require 200 ms to start integrating evidence, then what does a temporal threshold of 50 or 100 ms MD even mean? In contrast, in the urgent tasks there is no discrepancy: the behavioral discrimination time for the monkeys matches the neuronal differentiation time within a few milliseconds[4,8,9]. A case in point is the striking temporal correspondence between LIP activity and behavior found for the urgent color discrimination task (Fig. 6h).

The analysis based on rPT provides a uniquely precise view of how the subject's choice and the neural signals evolve jointly over time.

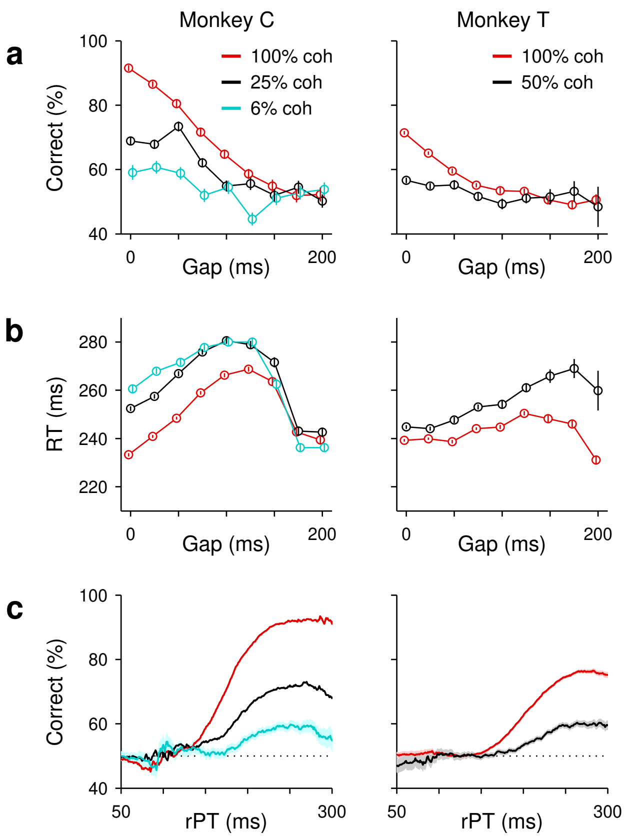

Supplementary Figure 1. Conventional psychophysical metrics in the CRDM task. Results are from the behavioral CRDM sessions, during which motion stimuli varied in their coherence level (colors; see legends). Data are from monkey C (left column; 9,544 trials for high coherence, 7,909 for intermediate, 5,375 for low) and monkey T (right column; 3,3971 trials for high coherence, 12,066 for intermediate). **a**, Psychometric curves showing choice accuracy as a function of gap. Performance varies as expected as a function of the two parameters that determine task difficulty: it decreases with increasing gap length, and it increases with increasing coherence. **b**, Mean RT as a function of gap. In general, response times increase with task difficulty, also as expected. The trend breaks down at longer gaps ($\geq$ 150 ms), during which most choices are guesses. **c**, Percentage of correct choices as a function of processing time, or tachometric curve. Error bars in all panels indicate $\pm$ 1 SE. Note that the gap = 0 condition, when the go signal and cue onset are simultaneous, is similar to a standard RT paradigm[2,6,10], albeit with high urgency to respond. The accuracy levels for gap = 0 in panel **a** are similar to the asymptotic performance levels in panel **c**, and are also comparable to the performance levels achieved in prior studies using short stimulus presentation times (of approximately 100–150 ms) under non-urgent conditions[3,11]. Importantly, though, performance under time pressure is dictated by rPT, as it varies much more sharply with rPT than with gap.

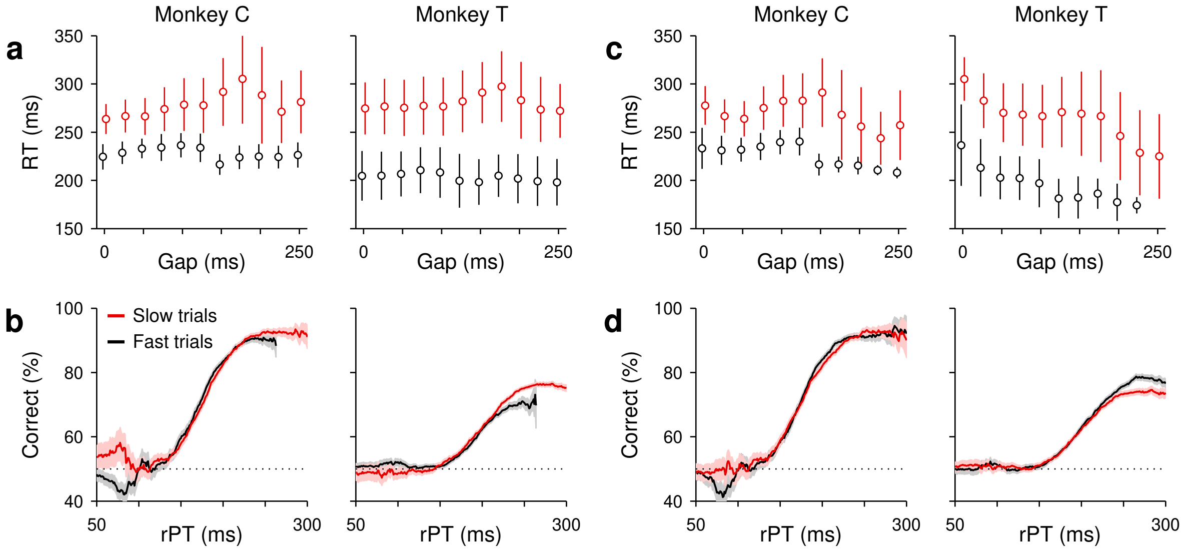

Supplementary Figure 2. Perceptual and motor performance are decoupled in the CRDM task. For each monkey, trials at 100% coherence were sorted into two groups, slow (red data) and fast (black data). These groups were defined in two ways. In Method 1, slow and fast trials were simply those with RTs above and below the overall median RT, respectively. In Method 2, trials were first sorted into non-overlapping rPT bins (20 ms width), and then the trials in each bin were split into slow and fast according to the median RT of that bin. **a**, **b**, Mean RT ± 1 SD as a function of gap (**a**) and percentage of correct choices ± 1 SE as a function of processing time, or tachometric curve (**b**), for the slow and fast trials obtained with Method 1. **c**, **d**, As in **a**, **b**, but for the slow and fast trials obtained with Method 2. All results are from the CRDM behavioral sessions; same 100% coherence data as in Fig. 1d. In spite of the large differences in RT, the fast and slow trials yielded tachometric curves that were largely indistinguishable. This shows that, under urgent conditions, perceptual performance (response accuracy) during motion discrimination can be reliably quantified independently of motor performance (response speed).

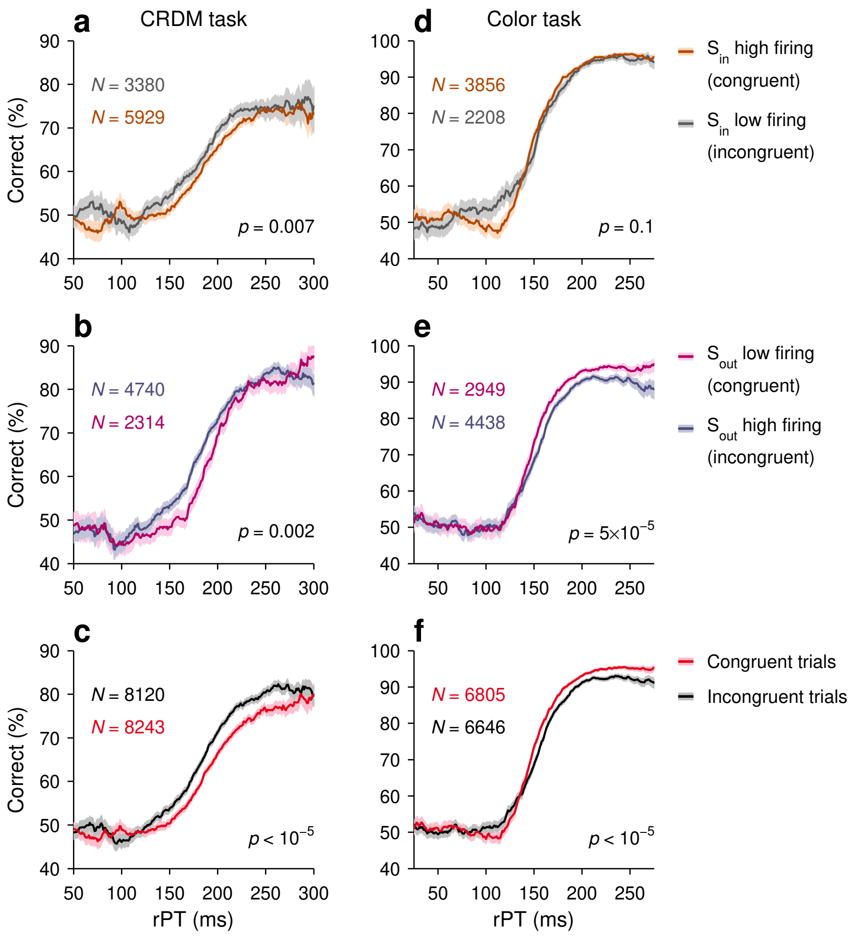

Supplementary Figure 3. Behavioral performance in the urgent tasks conditioned on LIP neuronal activity. **a**, Tachometric curves from all the CRDM trials in which the outcome was a saccade into the recorded cell's RF (S_{in}). For each neuron ($n = 51$), S_{in} trials were split according to whether the response was at or above the median (brown curve, high firing), or below the median (gray curve, low firing). The response was the spike count elicited in the 50 ms immediately preceding the onset of the saccade. High firing in S_{in} trials is congruent with a strong spatial signal, whereas low firing is incongruent. **b**, Tachometric curves from all the CRDM trials in which the outcome was a saccade away from the recorded cell's RF (S_{out}). For each neuron, S_{out} trials were split according to whether the response was at or above the median (blue curve, high firing), or below the median (purple curve, low firing). Low firing in S_{out} trials is congruent with a strong spatial signal, whereas high firing is incongruent. **c**, Results combining all congruent and incongruent trials across conditions. **d**–**f**, As in **a**–**c**, but for the urgent color discrimination task ($n = 56$). All traces show percent correct $\pm$ 1 SE within each rPT bin. Significance values shown are from resampling tests on the mean difference between each pair of curves (Methods). This difference was evaluated for rPTs of 130–230 ms for the CRDM data and for rPTs of 140–280 ms for the urgent color discrimination data (these ranges apply to all comparisons between conditioned curves in this and other figures). N indicates number of trials. Data in **c**, **f** are the same as in Fig. 3c, f. Note that the results are consistent between S_{in} and S_{out} conditions.

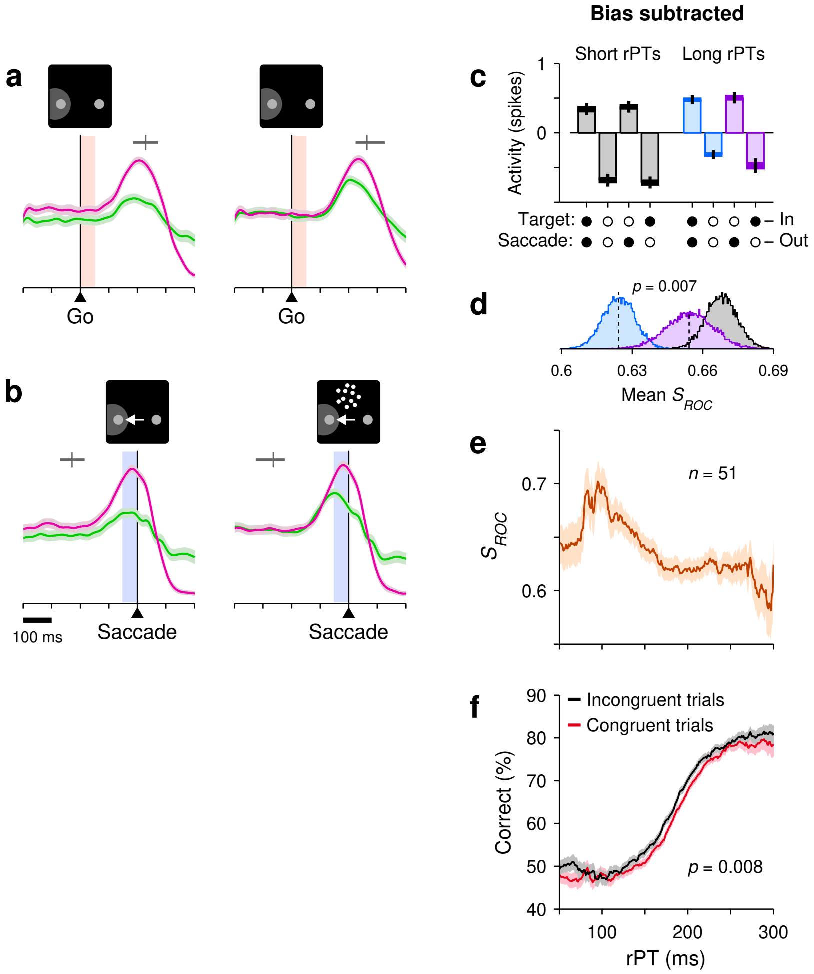

Supplementary Figure 4. An early choice bias does not account for the results in the CRDM task. **a**, Mean, normalized LIP activity recorded in the CRDM task (± 1 SE across cells; $n = 51$) aligned on the go signal. Colors indicate saccadic choices into (magenta) or away from the RF (green), with trials sorted into guesses (left, rPT $\leq$ 150 ms) and fully informed discriminations (right, rPT $\geq$ 200 ms). Gray crosses mark the saccade onset (90% range and median). To quantify the early bias in each trial, spikes were counted in the 50 ms time window immediately following the go signal (shaded areas). **b**, Mean, normalized LIP activity recorded in the CRDM task aligned on saccade onset. Same data as in Fig. 2e, f; same trials as in **a**, but aligned differently. Gray crosses mark the go signal (90% range and median). To quantify the presaccadic activity in each trial, spikes were counted in the 50 ms time window immediately preceding the saccade onset (shaded areas). **c–f**, Analysis results obtained after subtracting the early bias. The neural response in each trial was equal to the spike count from the standard presaccadic window (blue shade in **b**) minus the spike count from the earlier bias window (red shade in **a**). **c**, Mean centered responses sorted by outcome, as in Fig. 3a. **d**, Mean differential signal magnitudes for the three conditions in panel **c** indicated by color, as in Fig. 3b. **e**, Neuronal performance curve showing S_{ROC} (mean ± 1 SE across trials) as a function of rPT, as in Fig. 2h. **f**, Performance in the CRDM task conditioned on neuronal activity, as in Fig. 3c. The spatial signal based on the bias-subtracted spike counts demonstrated all the same trends found originally with the unaltered presaccadic responses.

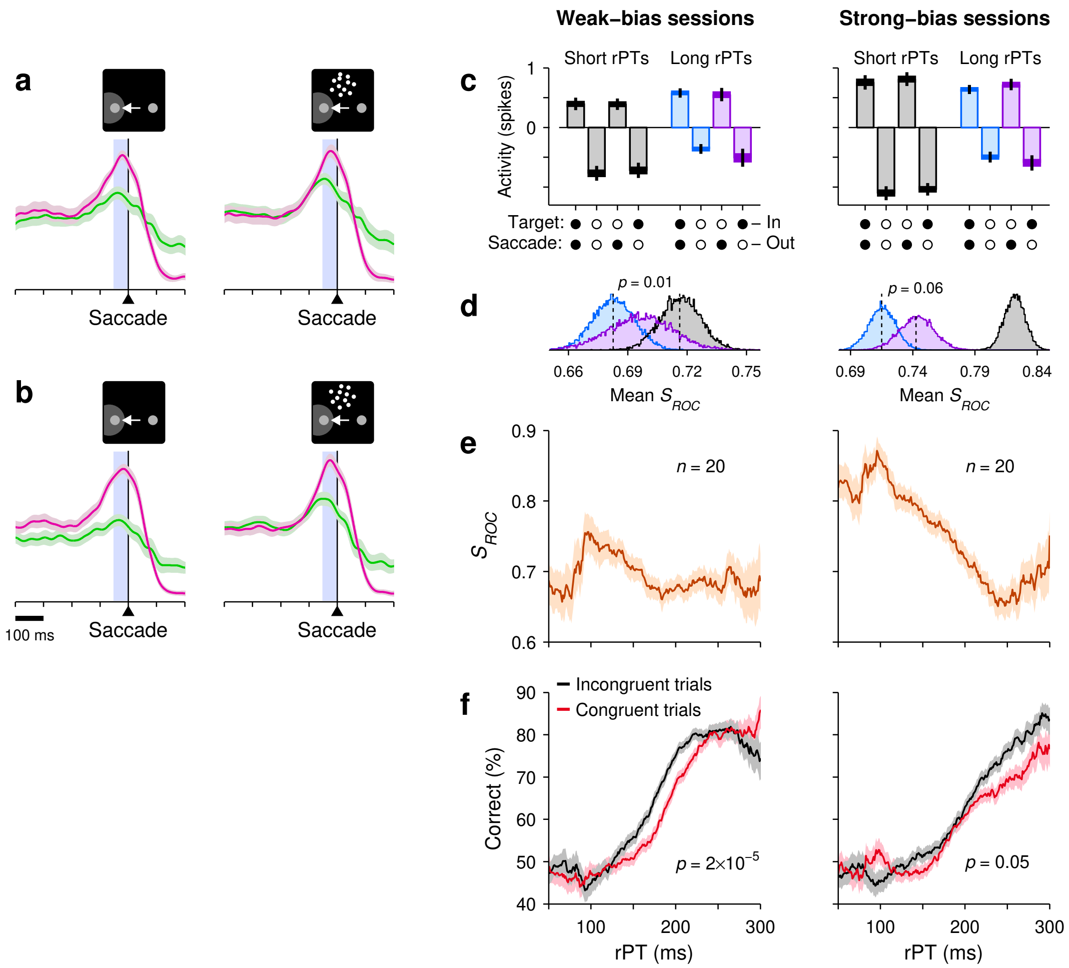

Supplementary Figure 5. The correlation between LIP activity and CRDM behavior is qualitatively the same for sessions in which a weak or a strong choice bias was observed. For each experimental session, the early bias was quantified by counting the spikes evoked in the 50 ms immediately following the go signal (Supplementary Fig. 4a, shaded areas) and calculating a spatial discrimination index (S_{ROC}) using all the spike counts from that session. Based on this index, the 20 sessions with the weakest bias and the 20 with the strongest were identified, and analyses were run separately for the two groups. **a**, Mean, normalized LIP activity as a function of time ($\pm$ 1 SE across cells; $n = 20$) in the sessions with the weakest early bias. Colors indicate saccadic choices into (magenta) or away from the RF (green), with trials sorted into guesses (left, rPT $\leq$ 150 ms) and fully informed discriminations (right, rPT $\geq$ 200 ms). **b**, As in **a**, but for the sessions with the strongest early bias. **c–f**, Analysis results for weak- (left column) and strong-bias sessions (right column). **c**, Mean centered responses sorted by outcome, as in Fig. 3a. **d**, Mean differential signal magnitudes for the three conditions in panel **c** indicated by color, as in Fig. 3b. **e**, Neuronal performance curves showing the presaccadic S_{ROC} (mean $\pm$ 1 SE across trials) as a function of rPT, as in Fig. 2h. **f**, Performance conditioned on neuronal activity, as in Fig. 3c. Although the magnitude of the spatial signal before the saccade did vary with the magnitude of the early bias, stronger presaccadic differentiation was still associated with shorter processing times and poorer performance, regardless of the bias.

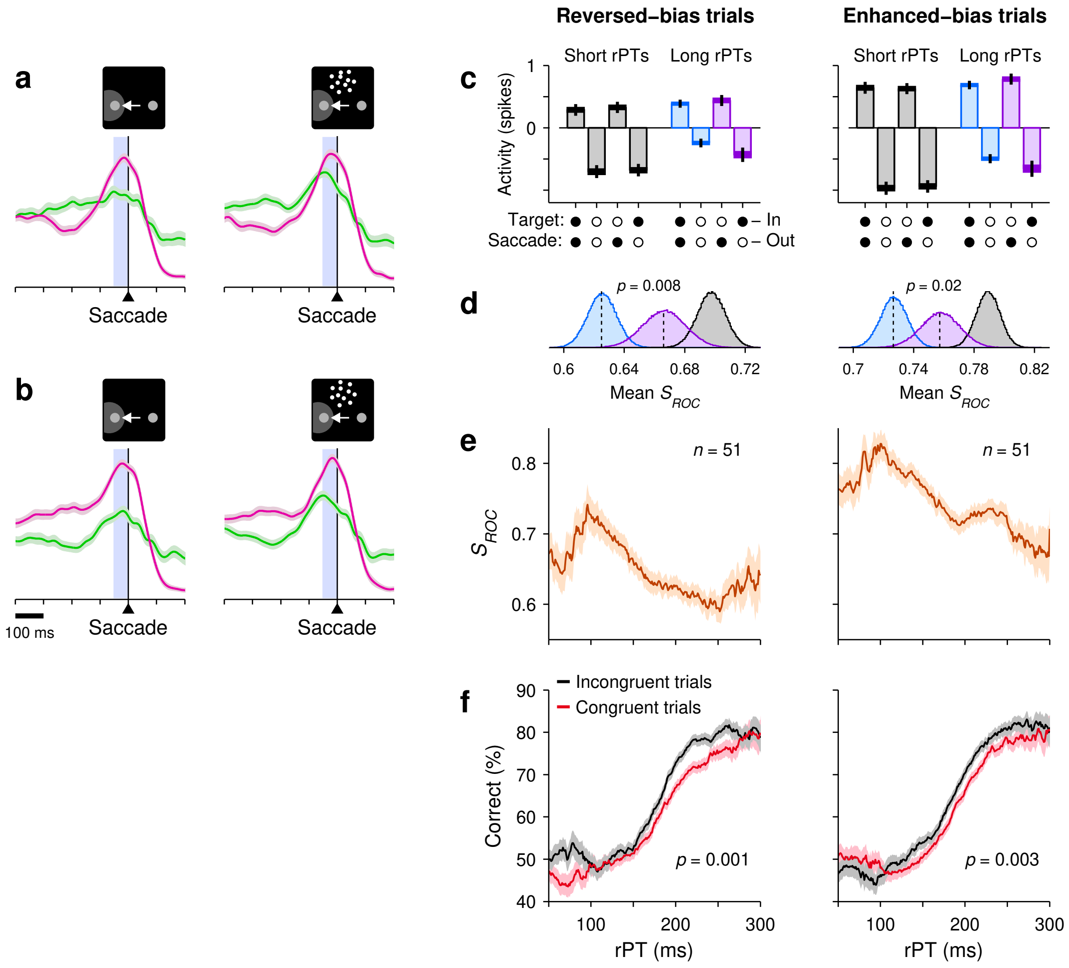

Supplementary Figure 6. Creating artifical biases through trial sorting does not change the observed correlation between LIP activity and CRDM behavior. For each recorded neuron, the early bias in each trial was quantified by counting the spikes evoked in the 50 ms immediately following the go signal (Supplementary Fig. 4a, shaded areas). Then, trials into and away from the RF were separately split into two groups according to the median spike count in the bias window, and the data for each of the 4 resulting groups were pooled across neurons. Finally, two such groups of trials (strong response in; weak response away) were paired to create a data set with an enhanced bias into the RF, and the other two groups (weak response in; strong response away) were paired to create a data set with a reversed bias, i.e., a bias away. Analyses were then run on the two halfs of the data thus parsed. **a**, Mean, normalized LIP activity as a function of time ($\pm$ 1 SE across cells; $n = 51$) for the data set with a reversed bias initially favoring the away direction. Colors indicate saccadic choices into (magenta) or away from the RF (green), with trials sorted into guesses (left, rPT $\leq$ 150 ms) and fully informed discriminations (right, rPT $\geq$ 200 ms). **b**, As in **a**, but for the data set with an enhanced early bias toward the RF. **c–f**, Analysis results for reversed- (left column) and enhanced-bias data sets (right column). Same format as in Supplementary Fig. 5c–f. Sorting trials in this fashion strongly alters the starting point of the evoked presaccadic responses, but the subsequent changes in activity maintain a consistent qualitative relationship with processing time and choice outcome regardless of that initial condition.

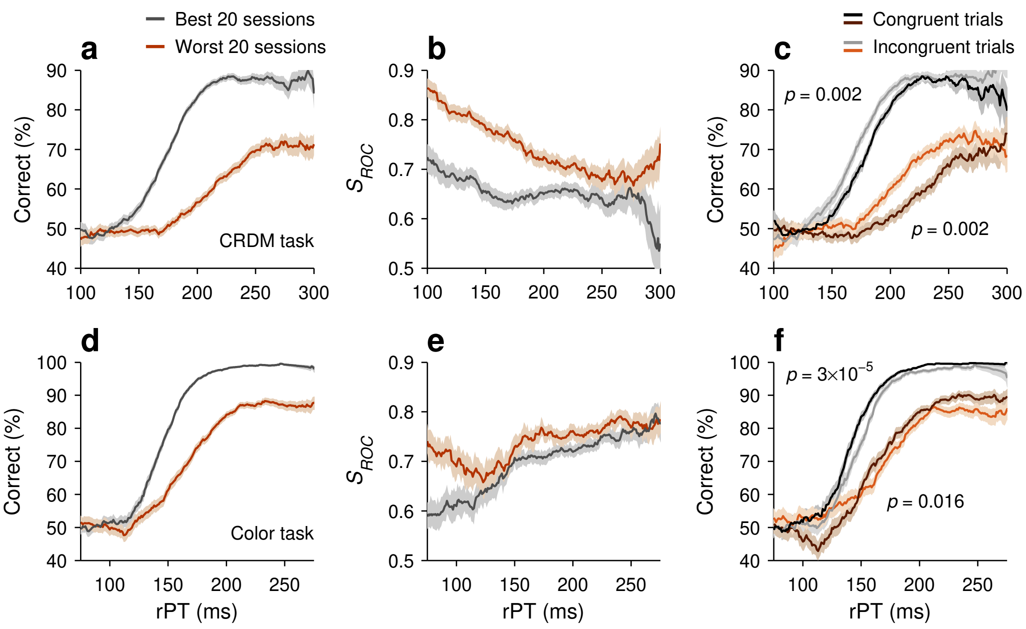

Supplementary Figure 7. Modulation of LIP activity during high- versus low-performance sessions. For both the motion- and color-based urgent tasks, recording sessions were ranked according to overall percent correct. The 20 sessions with the best performance (gray traces) and the 20 sessions with the poorest performance (brown traces) were selected, and analyses were run for each group separately. **a**, Tachometric curves for the best and worst CRDM sessions. As for other tachometric curves, traces show percentage of correct choices across trials in each rPT bin, and shaded error bands indicate $\pm$ 1 SE. **b**, Neurometric curves in the CRDM task, i.e., magnitude of presaccadic differentiation (mean $\pm$ 1 SE) as a function of processing time (as in Fig. 2h). In both groups of sessions, stronger LIP differentiation was associated with less evidence (shorter rPTs). **c**, Tachometric curves conditioned on neuronal activity in the CRDM task (as in Fig. 3c). In both groups of sessions, stronger LIP differentiation (congruent condition) was associated with worse perceptual performance. **d–f**, Same as **a–c**, but for the urgent color discrimination task (as in Figs. 6h and 3f). In this task, stronger LIP differentiation was associated with more evidence (longer rPTs; **e**) and improved perceptual performance (**f**). In each task, the relationship between the subjects' behavior and neuronal activity in LIP was similar for the two groups of sessions, in spite of the dramatically different performance levels.

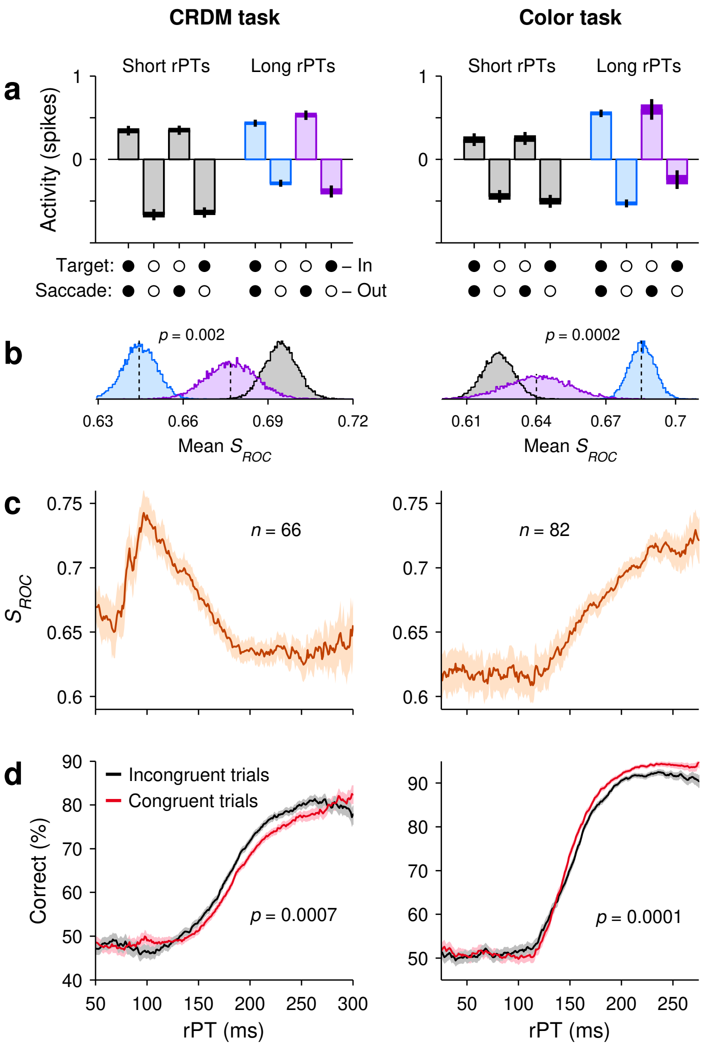

Supplementary Figure 8. Results with extended neuronal populations. The results in the main figures were based on neurons ($n = 51$ for the CRDM task, $n = 56$ for the urgent color discrimination task) that were fully characterized and satisfied several criteria: they had both visually-driven and saccade-related responses, and their RFs were well defined and consistent across tasks (Methods). This resulted in the exclusion of 15 additional neurons recorded in the CRDM experiment and 26 in the color-based. Here we show the results of analyses that included all the neurons recorded in the CRDM (left column, $n = 66$) and the urgent color discrimination task (right column, $n = 82$), with no exclusions. **a**, Mean, centered neuronal activity pooled across neurons and sorted by experimental condition (as in Fig. 3a, d). **b**, Distributions for mean presaccadic differentiation values (S_{ROC}) obtained by bootstrapping, for each of the three conditions above, as indicated by the corresponding colors (as in Fig. 3b, e). **c**, Neurometric curves, i.e., magnitude of presaccadic differentiation as a function of processing time (as in Figs. 2h, 6h). **d**, Behavioral performance conditioned on neuronal activity (as in Fig. 3c, f). Inclusion of the additional populations did not alter the results substantially.

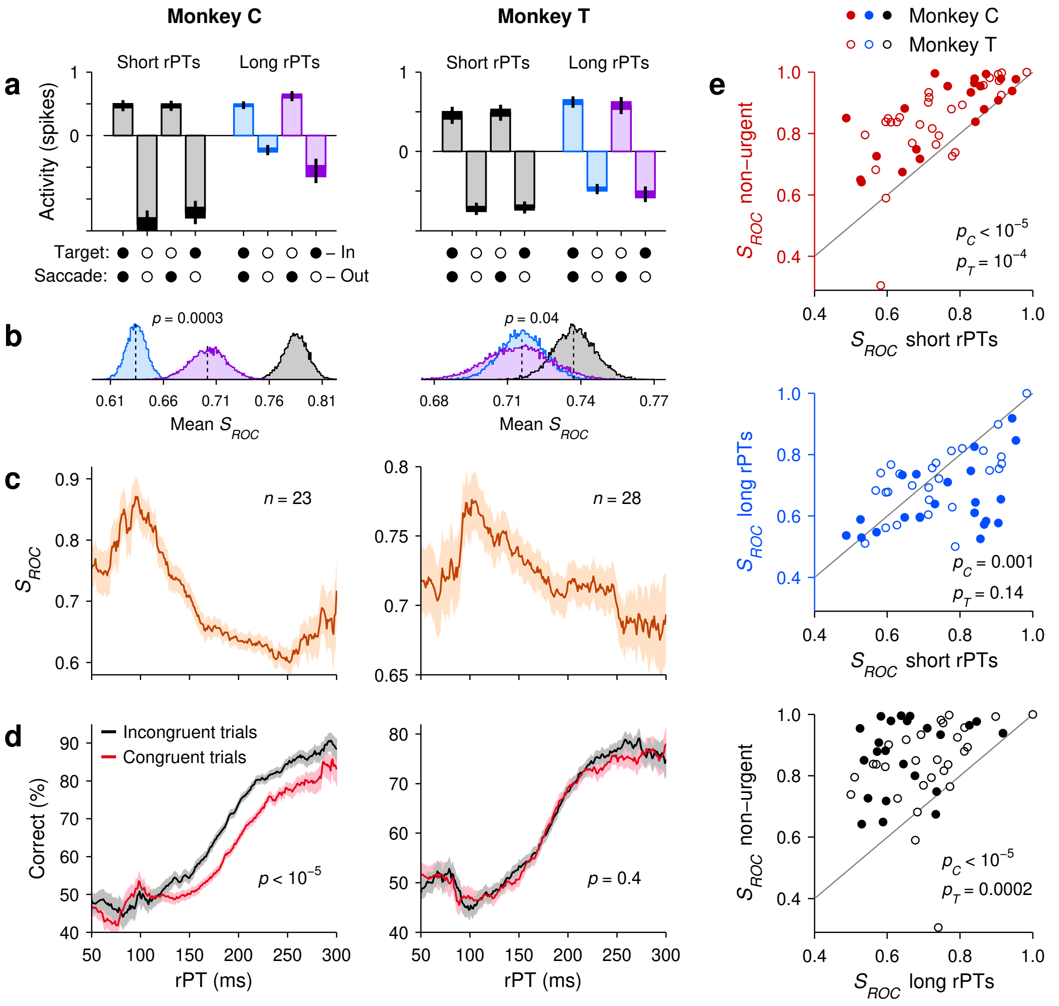

Supplementary Figure 9. Key results in the CRDM task computed separately for monkeys C (n = 23) and T (n = 28). **a**, Centered LIP activity during guesses (rPT $\leq$ 150 ms, gray) and informed choices (rPT $>$ 150 ms, blue, purple) sorted by experimental condition (as in Fig. 3a). **b**, Distributions for mean presaccadic differentiation values (S_{ROC}) for each of the three conditions above, as indicated by color (as in Fig. 3b). **c**, Neurometric curve, i.e., magnitude of presaccadic differentiation as a function of processing time (as in Fig. 2h). **d**, Behavioral performance conditioned on neuronal activity (as in Fig. 3c). **e**, Presaccadic S_{ROC} for individual neurons in the non-urgent RDM task, during guesses in the CRDM task (rPT $<$ 150 ms), and during fully informed choices in the CRDM task (rPT $\geq$ 200 ms). Data are as in Fig. 2g, but for each of the three pairwise comparisons shown separately. Significance values are for mean differences between conditions on the x and y axes (against zero; permutation tests) for each monkey.

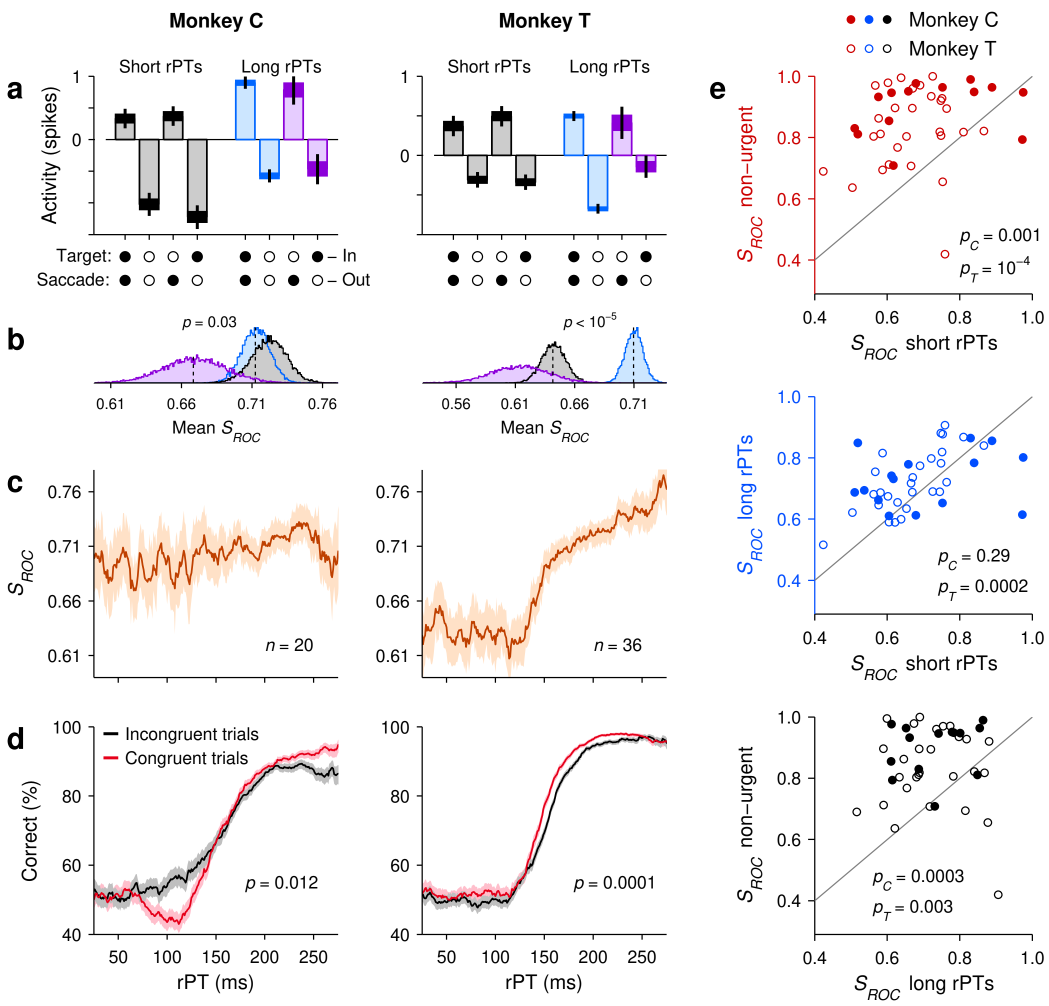

Supplementary Figure 10. Key results in the urgent color discrimination task computed separately for monkeys C ($n = 20$) and T ($n = 36$). **a**, Centered LIP activity during guesses (rPT $\leq$ 125 ms, gray) and informed choices (rPT $>$ 125 ms, blue, purple) sorted by experimental condition (as in Fig. 3d). **b**, Distributions for mean presaccadic differentiation values (S_{ROC}) for each of the three conditions above, as indicated by color (as in Fig. 3e). **c**, Neurometric curve, i.e., magnitude of presaccadic differentiation as a function of processing time (as in Fig. 6h). **d**, Behavioral performance conditioned on neuronal activity (as in Fig. 3f). **e**, Presaccadic S_{ROC} for individual neurons in the non-urgent color discrimination task, during guesses in the urgent version (rPT $<$ 125 ms), and during fully informed choices in the urgent version (rPT $\geq$ 175 ms). Data are as in Fig. 6g, but for each of the three pairwise comparisons shown separately. Significance values are for mean differences between conditions on the x and y axes (against zero; permutation tests) for each monkey.

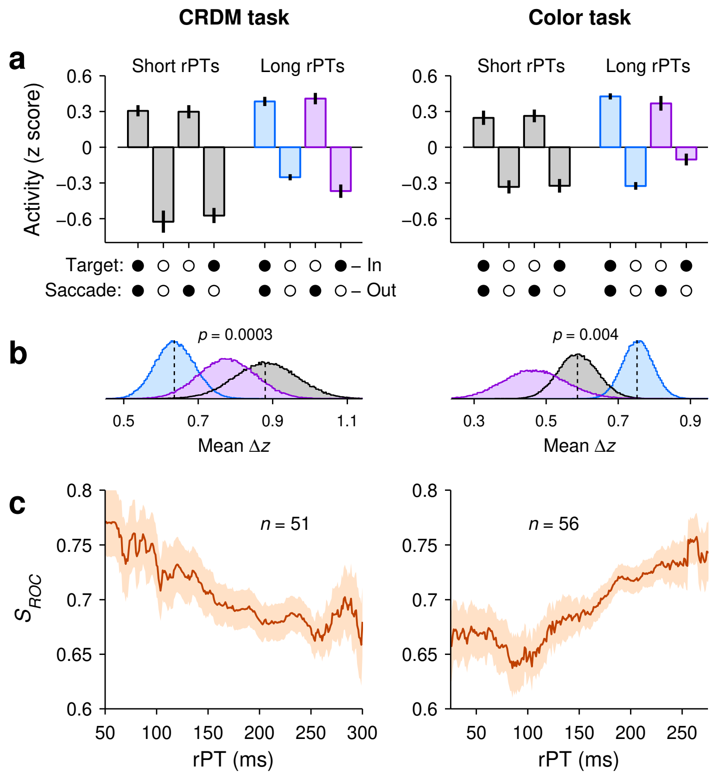

Supplementary Figure 11. Alternate procedure for combining the data across neurons. In the main figures (Figs. 2h, 6h, 3a, d), we first pooled all the spike counts from all the neurons, separately for saccades into (S_{in}) and away from the RF (S_{out}), and then quantified the separation between the two resulting distributions by computing the S_{ROC} (Methods; Supplementary Fig. 14). Here we present an alternate analysis in which S_{in} and S_{out} conditions are first contrasted for each neuron and then the results are averaged across neurons. Results are for the CRDM (left column, $n = 51$) and the urgent color discrimination task (right column, $n = 56$). **a**, LIP activity during guesses and informed choices sorted by outcome (same short- and long-rPT intervals as in Fig. 3a, d). Activity corresponds to presaccadic spike counts (same as in other analyses) that were z-scored for each neuron, sorted, and then averaged across neurons. Data are mean $\pm$ 1 SE across cells. **b**, Differential signal magnitudes for the three conditions in **a** indicated by color. Here, the differential signal of each cell is the mean difference between the z-scored responses in S_{in} and S_{out} conditions. Thus, the mean Δz is equal to $\langle z_{IN} - z_{OUT} \rangle$, where the brackets indicate an average over neurons. Curves are bootstrapped distributions. Sigificance is from a one-sided paired resampling test (Methods). **c**, Mean neurometric curve, i.e., magnitude of presaccadic differentiation as a function of processing time. In this case, a neurometric curve was first computed for each individual neuron (bin width $=$ 81 ms) and then the results were averaged over neurons. Shades indicate $\pm$ 1 SE across cells. The results of these analyses are more variable than those in the main figures, but show the same qualitative trends for how spatial discriminability depends on processing time and trial outcome in each task.

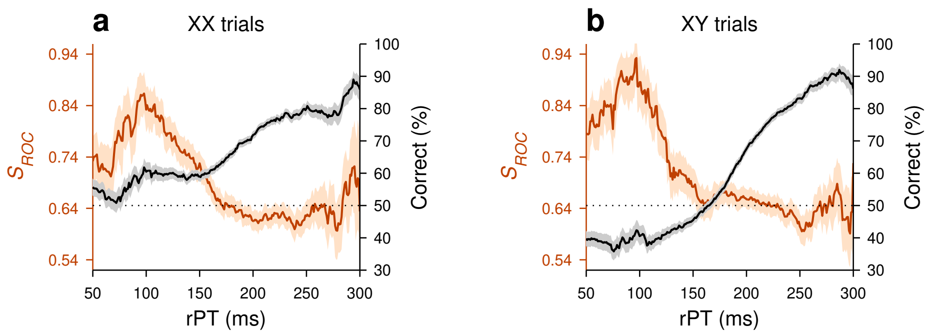

Supplementary Figure 12. History effects in the CRDM task. In general, in our experiments, performance on a given trial was minimally affected by the stimuli and choices that occurred before that trial. The graphs show the strongest effect found, which was present in only one subject, monkey C. It depended on target location, i.e., motion direction. Each panel compares the neurometric (brown trace) and the tachometric curve (black trace) from the same trials, as in Figs. 2h, 6h, but having selected those trials based on prior history. **a**, Data from XX trials, in which the target location in the current trial was the same as in the previous trial. The horizontal dotted line marks chance performance (50% correct). **b**, Data from XY trials, in which the target location switched from that in the previous trial. Note that guesses (rPT $\leq$ 150 ms, black traces) are biased toward the prior target location, as indicated by their offset from chance, but the LIP differentiation shows qualitatively similar patterns regardless of history.

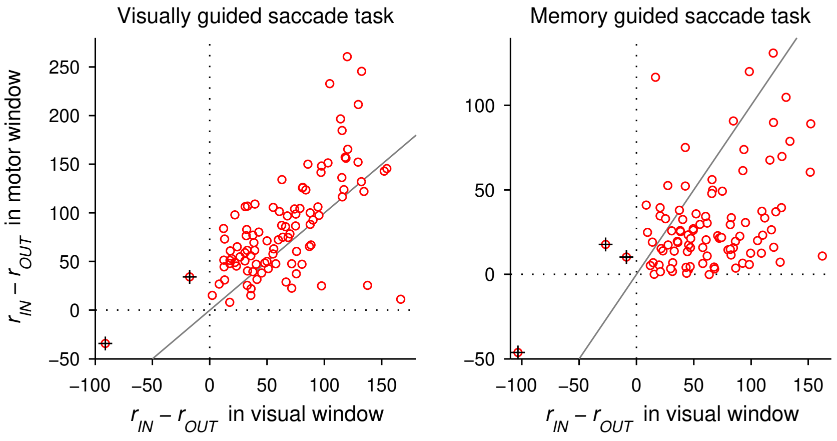

Supplementary Figure 13. Alignment between visual and motor RFs. Data are from 101 LIP neurons that comprise the majority of those that were considered in the analyses of the two urgent tasks (101 of 107). Each data point represents one neuron recorded in the visually guided (left panel) or the memory guided saccade task (right panel). The RFs of these units were defined according to their preference in the urgent tasks. Values on the x axes indicate mean differential activity in a visual window (50:120 ms, aligned on target onset), and contrast the response evoked by a stimulus appearing in the RF (r_{IN}) versus the diametrically opposite location (r_{OUT}). Values on the y axes indicate mean differential activity in a motor window (-70:0 ms, aligned on saccade onset), and contrast the response evoked just prior to a saccade into the RF (r_{IN}) versus to the diametrically opposite location (r_{OUT}). Units are spikes/s. For most neurons, the numbers on the x and y axes are both positive (black crosses mark exceptions). This means that (1) the cells overwhelmingly had the same spatial preference for their visual and movement-related responses, and (2) such preference was consistent between the urgent and single-target tasks.

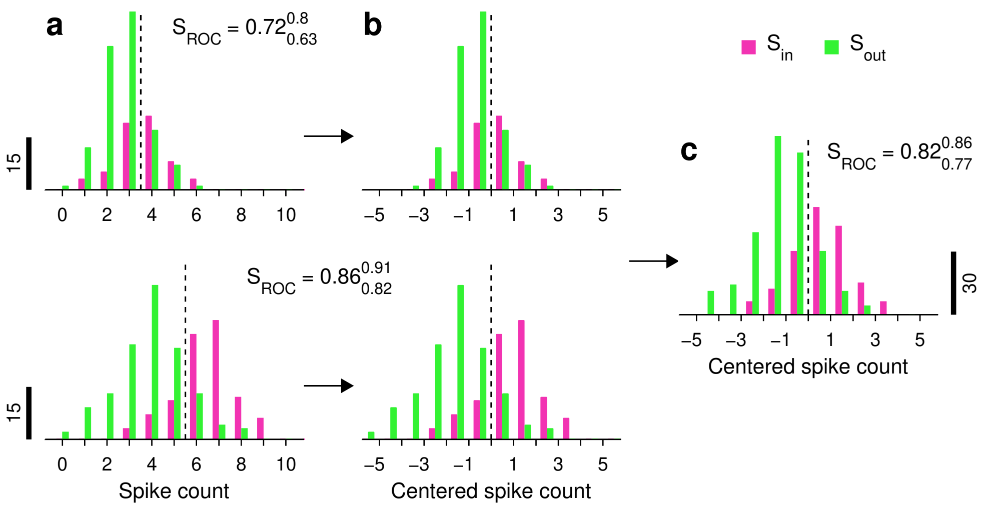

Supplementary Figure 14. Procedure for pooling the data across neurons and computing the average magnitude of their spatial signal. This example illustrates the pooling method for two neurons recorded in the CRDM task. For each cell, the response in each trial was the spike count collected in the 50 ms immediately preceding saccade onset. Responses were sorted by condition, for trials in which the saccade was into the RF (S_{in}, magenta bars) and for trials in which the saccade was away (S_{out}, green bars). In this case all rPTs are included. **a**, Spike count histograms from two LIP neurons (top and bottom). For each cell, the dashed line is the value (θ) intermediate between the mean spike counts for S_{in} and S_{out} trials. The magnitude of the differential response based on each cell's data is indicated, with 95% confidence limits (from bootstrap). **b**, Same data as in **a** but centered, i.e., after subtracting θ from each spike count. Individual S_{ROC} values do not change, as they are invariant to linear transformations of the data. **c**, Histograms for the pooled, centered data from the two neurons. Note that the resulting S_{ROC}, which is computed exactly as for the single cells, is intermediate between their values.

Supplementary References

1. Wickelgren, W. A. Speed-accuracy tradeoff and information processing dynamics. Acta Psychol. **41**, 67–85 (1977).
2. Palmer, J., Huk, A. C. & Shadlen, M. N. The effect of stimulus strength on the speed and accuracy of a perceptual decision. J. Vis. **5**, 376–404 (2005).
3. Kiani, R., Hanks, T. D. & Shadlen, M. N. Bounded integration in parietal cortex underlies decisions even when viewing duration is dictated by the environment. J. Neurosci. **28**, 3017–3029 (2008).
4. Stanford, T. R. & Salinas, E. Urgent decision making: resolving visuomotor interactions at high temporal resolution. Annu. Rev. Vis. Sci. **7**, 323–348 (2021).
5. Shadlen, M. N. & Newsome, W. T. Neural basis of a perceptual decision in the parietal cortex (area LIP) of the rhesus monkey. J. Neurophysiol. **86**, 1916–1936 (2001).
6. Roitman, J. D. & Shadlen, M. N. Response of neurons in the lateral intraparietal area during a combined visual discrimination reaction time task. J. Neurosci. **22**: 9475–9489 (2002).
7. de Lafuente, V., Jazayeri, M. & Shadlen, M. N. Representation of accumulating evidence for a decision in two parietal areas. J. Neurosci. **35**, 4306–4318 (2015).
8. Stanford, T. R., Shankar, S., Massoglia, D. P., Costello, M. G. & Salinas, E. Perceptual decision making in less than 30 milliseconds. Nat. Neurosci. **13**, 379–385 (2010).
9. Costello, M. G., Zhu, D., Salinas, E. & Stanford, T. R. Perceptual modulation of motor — but not visual — responses in the frontal eye field during an urgent-decision task. J. Neurosci. **33**, 16394–16408 (2013).
10. Huk, A. C. & Shadlen, M. N. Neural activity in macaque parietal cortex reflects temporal integration of visual motion signals during perceptual decision making. J. Neurosci. **25**, 10420–1036 (2005).
11. Gold, J. I. & Shadlen, M. N. The influence of behavioral context on the representation of a perceptual decision in developing oculomotor commands. J. Neurosci. **23**, 632–651 (2003).
