# Supplementary material for: A conflict between spatial selection and evidence accumulation in area LIP
Source: Nat Commun. 2022 Aug 1;13:4463. doi: 10.1038/s41467-022-32209-z (PMC9343639; doi:10.1038/s41467-022-32209-z)
Supplement: Supplementary file 3 — Reporting Summary [file 41467_2022_32209_MOESM3_ESM.pdf]

Corresponding author(s): Emilio Salinas

Last updated by author(s): Jun 7, 2022

Reporting Summary

Nature Portfolio wishes to improve the reproducibility of the work that we publish. This form provides structure for consistency and transparency in reporting. For further information on Nature Portfolio policies, see our Editorial Policies and the Editorial Policy Checklist.

Statistics

For all statistical analyses, confirm that the following items are present in the figure legend, table legend, main text, or Methods section.

n/a	Confirmed	
☐	☒	The exact sample size (*n*) for each experimental group/condition, given as a discrete number and unit of measurement
☐	☒	A statement on whether measurements were taken from distinct samples or whether the same sample was measured repeatedly
☐	☒	The statistical test(s) used AND whether they are one- or two-sided<br>*Only common tests should be described solely by name; describe more complex techniques in the Methods section.*
☐	☒	A description of all covariates tested
☐	☒	A description of any assumptions or corrections, such as tests of normality and adjustment for multiple comparisons
☐	☒	A full description of the statistical parameters including central tendency (e.g. means) or other basic estimates (e.g. regression coefficient) AND variation (e.g. standard deviation) or associated estimates of uncertainty (e.g. confidence intervals)
☐	☒	For null hypothesis testing, the test statistic (e.g. *F*, *t*, *r*) with confidence intervals, effect sizes, degrees of freedom and *P* value noted<br>*Give P values as exact values whenever suitable.*
☒	☐	For Bayesian analysis, information on the choice of priors and Markov chain Monte Carlo settings
☒	☐	For hierarchical and complex designs, identification of the appropriate level for tests and full reporting of outcomes
☐	☒	Estimates of effect sizes (e.g. Cohen's *d*, Pearson's *r*), indicating how they were calculated

Our web collection on statistics for biologists contains articles on many of the points above.

Software and code

Policy information about availability of computer code

Data collection	For sessions in which dot-motion tasks were performed, all gaze-contingent stimulus presentation and reward delivery were controlled using Psychtoolbox (Brainard, 1997; Kleiner et al., 2007) version 2.0 (publicly available); for all other sessions, gaze-contingent stimulus presentation and reward delivery were controlled via a custom-designed PC-based software package from Ryklin Software (2016 version).
Data analysis	All analyses were performed with Matlab release R2013b or more recent.

For manuscripts utilizing custom algorithms or software that are central to the research but not yet described in published literature, software must be made available to editors and reviewers. We strongly encourage code deposition in a community repository (e.g. GitHub). See the Nature Portfolio guidelines for submitting code & software for further information.

Data

Policy information about availability of data

All manuscripts must include a data availability statement. This statement should provide the following information, where applicable:

- Accession codes, unique identifiers, or web links for publicly available datasets
- A description of any restrictions on data availability
- For clinical datasets or third party data, please ensure that the statement adheres to our policy

The behavioral and presaccadic spike-count data that support the findings of this study are publicly available from the Zenodo repository, https://doi.org/10.5281/zenodo.6604002.
Source data are provided with this paper.

Field-specific reporting

Please select the one below that is the best fit for your research. If you are not sure, read the appropriate sections before making your selection.

☒ Life sciences ☐ Behavioural & social sciences ☐ Ecological, evolutionary & environmental sciences

For a reference copy of the document with all sections, see nature.com/documents/nr-reporting-summary-flat.pdf

Life sciences study design

All studies must disclose on these points even when the disclosure is negative.

Sample size	Neuron sample sizes (~50 cells per experiment) were chosen based on the consistency of the functional properties of the recorded neurons and based on comparable prior work (Stanford et al., Nature Neuroscience 13:379, 2010; Scerra et al., Current Biology 29:294, 2019; Sarno et al., PNAS 119:e2113311119, 2022). The use of two subjects is standard for recordings studies in nonhuman primates (Mante et al., Nature 503:78, 2013; Hagan and Pesaran, Nature 604:708, 2022) .
Data exclusions	Some neurons that were recorded and fully characterized were excluded from the studied samples based on conventional functional criteria, namely, they had no significant visual or memory activity in the single-target tasks; they were not significantly activated presaccadically; or their spatial preference for contralateral/ipsilateral stimuli either was ambiguous or clearly flipped between different tasks.
Replication	Data were analyzed separately for each monkey; for neurons grouped according to different criteria; and for experimental sessions according to behavioral performance. Analyses were double checked with different quantitative methods, and consistency of statistical results was verified by applying alternative tests (e.g., parametric and non-parametric) to the same data sets. No inconsistencies in replication were found.
Randomization	Sample randomization was not applicable to the experimental design, as data were not allocated by predefined groups.
Blinding	Blinding was not applicable to the experimental design, as data were not allocated by predefined groups.

Reporting for specific materials, systems and methods

We require information from authors about some types of materials, experimental systems and methods used in many studies. Here, indicate whether each material, system or method listed is relevant to your study. If you are not sure if a list item applies to your research, read the appropriate section before selecting a response.

Materials & experimental systems

n/a	Involved in the study	
☒	☐	Antibodies
☒	☐	Eukaryotic cell lines
☒	☐	Palaeontology and archaeology
☐	☒	Animals and other organisms
☒	☐	Human research participants
☒	☐	Clinical data
☒	☐	Dual use research of concern

Methods

n/a	Involved in the study	
☒	☐	ChIP-seq
☒	☐	Flow cytometry
☒	☐	MRI-based neuroimaging

Animals and other organisms

Policy information about studies involving animals; ARRIVE guidelines recommended for reporting animal research

Laboratory animals	Subjects were two male, adult, rhesus monkeys, Macacca mulatta. They were 4.7 and 5.7 years of age at the start of the experiment.
Wild animals	No wild animals were used in this study.
Field-collected samples	No field-collected samples were used in this study.
Ethics oversight	All experimental procedures were conducted in accordance with NIH guidelines and USDA regulations, and were approved by by the Institutional Animal Care and Use Committee (IACUC) of Wake Forest School of Medicine.

Note that full information on the approval of the study protocol must also be provided in the manuscript.
